# Muscle or Fascial System Lesion (Part I): Understanding the Continuum From Micro to Macro From a Clinical Perspective

**DOI:** 10.7759/cureus.93465

**Published:** 2025-09-29

**Authors:** Saverio Colonna, Fabio Casacci, Paolo MInafra

**Affiliations:** 1 Rehabilitation Medicine, Spine Center, Bologna, ITA; 2 Research and Development, Osteopathic Spine Center Education, Bologna, ITA; 3 Radiology, Affidea Modena, Bologna, ITA

**Keywords:** aponeurosis, elastic energy storage, fascial system, intramuscular connective tissue (imct), muscle injury, muscle strain, myoaponeurotic injury, myoconnective junction, myotendinous junction (mtj), sports medicine

## Abstract

Muscle injuries are the most common traumatic events in both team and individual sports, representing a significant cause of time lost from training and competition. Traditionally described as “muscle tears,” these injuries have been interpreted almost exclusively from a contractile perspective. However, emerging evidence highlights the critical role of the myoconnective architecture, comprising the intramuscular connective tissue (endomysium, perimysium, and epimysium), aponeuroses, basal lamina, and extracellular matrix, in force transmission, injury susceptibility, and tissue repair.

This narrative review integrates anatomical, histological, and biomechanical evidence to describe skeletal muscle as a composite unit in which passive connective structures function synergistically with active contractile elements. Particular attention is given to the role of the myoaponeurotic and myofascial junctions, frequent sites of indirect, stretch-related injuries, and to the elastic properties of aponeurotic and fascial tissues in energy storage and dissipation during locomotion.

Understanding how microscopic organization influences macroscopic injury mechanisms reframes muscle injury as a lesion primarily affecting the myoconnective interfaces rather than isolated muscle fibers. This paradigm shift has implications for diagnosis, prognosis, and rehabilitation, emphasizing the need for preventive and therapeutic strategies that target both contractile and connective components.

Part II of this series will apply this framework to the medial gastrocnemius lesion (“tennis leg”), illustrating how detailed knowledge of myoconnective anatomy informs clinical decision-making in sports medicine.

## Introduction and background

Muscle injuries represent the most frequent type of trauma in both team and individual sports and are the leading cause of absence from training sessions and competitions [1-3]. In soccer, in particular, they account for approximately half of all recorded injuries [2]. The four most commonly affected muscle groups are well established [4,5], among which the hamstrings are the most vulnerable [6,7], followed by the adductors, the rectus femoris, and the calf muscles.

Muscle injuries represent a significant clinical and economic burden in sports medicine, not only due to their high incidence but also because of the frequent risk of recurrence and prolonged recovery times. Despite continuous advances in diagnosis and rehabilitation, these injuries remain a leading cause of time lost from training and competition, highlighting the need for a deeper understanding of their underlying mechanisms.

Traditionally, the concept of “muscle tear” has been associated almost exclusively with damage to the contractile elements of the muscle fibers. However, this perspective does not fully capture the complex anatomical and functional integration between muscle fibers and their surrounding connective tissue.

Emerging evidence suggests that structures such as the basal lamina, endomysium, perimysium, epimysium, and aponeuroses play a crucial role in force transmission, energy storage, and vulnerability to injury. This broader view reframes muscle trauma as a lesion involving the myoconnective system rather than the contractile apparatus alone.

The aim of this narrative review is therefore to integrate anatomical, histological, and biomechanical data to provide a comprehensive framework for understanding muscle injuries as multifaceted lesions of the myoconnective architecture.

In this first part, we describe the organization of skeletal muscle from micro to macro and highlight the structural continuum that links contractile and connective components. In Part II, this conceptual approach will be applied to the medial gastrocnemius lesion (“tennis leg”), offering an example of how this perspective can inform clinical reasoning and rehabilitation strategies.

## Review

Commonly used classifications (ISMuLT, Munich, and BAMIC)

Several classifications of muscle injuries exist, including the Munich Muscle Injury Classification [8], the ISMuLT classification [6], and the British Athletics Classification [9]. The systematic adoption of these classifications could help improve the diagnosis, prognosis, and clinical management of muscle injuries (Table 1).

**Table 1:** Comparison of muscle injury classifications The table summarizes the main features of the three most commonly used classification systems for muscle injuries: ISMuLT, Munich, and BAMIC. BAMIC: British Athletics Muscle Injury Classification, MRI: magnetic resonance imaging, DOMS: delayed-onset muscle soreness

Classification	Basis	Key features	Clinical use
ISMuLT	Mechanism (direct vs. indirect) and structural involvement	Direct: external trauma, indirect: stretching overload, subdivision: structural vs. non-structural	Guides diagnosis and prognosis, integrates the mechanism
Munich	Clinical mechanism-based system	Functional (non-structural): fatigue-related or DOMS, structural: partial tears, complete tears, contusions	Widely adopted in sports medicine for on-field classification
BAMIC	MRI-based (size and location of lesion)	Grades 0–4 based on MRI, grades 1–3 subdivided by location: (a) myofascial, (b) myotendinous, and (c) tendinous	Supports imaging-based staging and prognostic planning

According to the classification proposed by ISMuLT [6], muscle injuries are divided into direct and indirect types, depending on the traumatic mechanism. Indirect injuries are further subdivided into structural and non-structural. External forces generally cause direct injuries, whereas indirect injuries result from a stretching mechanism provoked by a sudden and forceful elongation beyond the viscoelastic limits of the muscle during intense contraction [10].

Indirect structural muscle injuries, commonly known as “muscle tears,” are the most frequently observed in daily clinical practice in sports traumatology and represent the greatest rehabilitative challenge, as the traumatic event is more likely to recur and currently lacks standardized, safe therapeutic strategies [11].

The British Athletics Muscle Injury Classification (BAMIC) [9] is an MRI-based system with five levels that considers both the severity and the location of the injury. Although it was developed primarily for injuries of the posterior thigh muscles, it is also used for other muscle injuries. Grades 1-3 are further subdivided based on location: (a) myofascial (peripheral), (b) myotendinous/muscular junction, and (c) tendinous.

Need for terminological precision: muscular versus myofascial

Paraphrasing the title of a narrative review by Stecco et al., “Fascial or Muscle Stretching?” [12], the title of this article aims to emphasize a question that is appropriate to ask in light of recent research [13-15]: when we speak of muscle injury, are we actually describing the true anatomical and pathological picture of the traumatic event?

That there is a genuine need to better define the exact site of the injury can be inferred from the title of a recent article: “Is there a need to reconsider the importance of myoaponeurotic injury within the nomenclature of sports-related muscle injury?”, in which the authors [16] highlight the importance of clarity regarding the precise terminology of the connective tissues that are part of the muscle structure.

Although there is some debate in the anatomical literature about the precise definition of fascia, it is generally accepted that fascia, aponeurosis, and tendons represent a continuum of musculoskeletal connective tissues, characterized by a progressive increase in thickness and regularity of collagen fiber orientation (tendons > aponeuroses > fascia) [16]. Similar to tendons, but unlike fascia, aponeuroses exhibit more regularly oriented fibers, reflecting their role in resisting predominantly unidirectional tensile forces [16].

Anatomical review of skeletal muscle: from micro to macro

Skeletal muscle is primarily composed of muscle cells and the connective tissue that surrounds them, organized in a highly structured manner. Like any organ, it also contains small-caliber blood vessels, capillaries, neural innervation, and variable amounts of immune cells [17,18].

Myofiber and sarcomere: organization and contractile function

Skeletal muscle cells are referred to as myofibers due to their elongated shape; they are cylindrical, striated, and multinucleated, and their primary function is the production of contractile force for locomotion [19] and for body movement in general.

The length of skeletal muscle fibers in humans ranges from a few millimeters (stapedius muscle) to approximately 50 cm (sartorius muscle). At the same time, their diameter can vary from 15-20 μm (extrinsic eye muscles) to over 100 μm in trained power athletes [17].

The average mass of the medial gastrocnemius depends on age, sex, and training status and ranges from 150-250 g [20]. Assuming an average cross-sectional diameter of 100 μm per fiber and a known fiber density of approximately 2.5-3.5 million fibers per kilogram of muscle [21], a 200 g medial gastrocnemius would contain approximately 500,000-700,000 myofibers. For the hamstrings (biceps femoris (BFlh), semitendinosus, and semimembranosus), which have a combined mass of approximately 500-700 g in untrained adults [20], applying the same fiber density yields approximately 1.5-2 million total myofibers.

Strength training primarily induces hypertrophy by increasing myofiber cross-sectional area (diameter), whereas hyperplasia (an increase in fiber number) is minimally evident in humans and is not considered a major contributor to muscle growth [22,23].

The sarcomere is the contractile unit of the myofiber and generates contraction and shortening through the sliding of actin and myosin filaments. The series connection of multiple sarcomeres is called a "myofibril." Each muscle fiber can contain several thousand myofibrils.

The number of sarcomeres contained within a myofibril depends mainly on the length of the muscle fiber and the average length of a sarcomere. The average resting sarcomere length is approximately 2.2 μm (range: 2.0-2.5 μm). Fiber length varies by muscle: small muscles (e.g., stapedius) measure only a few millimeters; the medial gastrocnemius is about 3-6 cm; the hamstrings are approximately 20-30 cm; and long muscles (e.g., sartorius) can reach up to 50 cm.

Calculated as the number of sarcomeres = fiber length (μm) / sarcomere length (μm), the sartorius has approximately 227,000 sarcomeres, the hamstrings have 114,000 sarcomeres, and the medial gastrocnemius has 23,000 sarcomeres.

Each myofiber is a syncytium with sarcomeres arranged in series, in the construction of myofibrils, along its length. In pennate muscles (e.g., gastrocnemius), fibers are shorter and contain fewer sarcomeres in series but more fibers in parallel. In fusiform muscles (e.g., sartorius), fibers are long and contain many sarcomeres in series, favoring excursion.

Sarcolemma and basal lamina

Each myofiber possesses specific molecular chains, such as integrins and dystrophin, which connect the contractile apparatus of actin and myosin filaments to the extracellular matrix (ECM) through the sarcolemma. Integrins are largely localized at the myotendinous junction (MTJ). Sarcomeric actin binds to the β1 subunit of the muscle-specific integrin α7β1 [17] via a complex of molecules situated in the subsarcolemmal region; this integrin, in turn, connects to ECM proteins [24]. The molecules of the dystrophin-associated complex are distributed along the entire sarcolemma, but are particularly concentrated at both the MTJ and the neuromuscular junction.

Following this arrangement, the shortening tension generated by the sarcomeres is not transmitted outward by stretching the sarcolemma itself, but rather through bridges that cross the sarcolemma and transfer the force directly to the basal lamina, which is intimately connected to the endomysium. In this complex, actin binds to dystrophin, which in turn interacts with three protein complexes: dystroglycans, sarcoglycans, and syntrophins, all of which span the sarcolemmal membrane [25].

**Figure 1:**
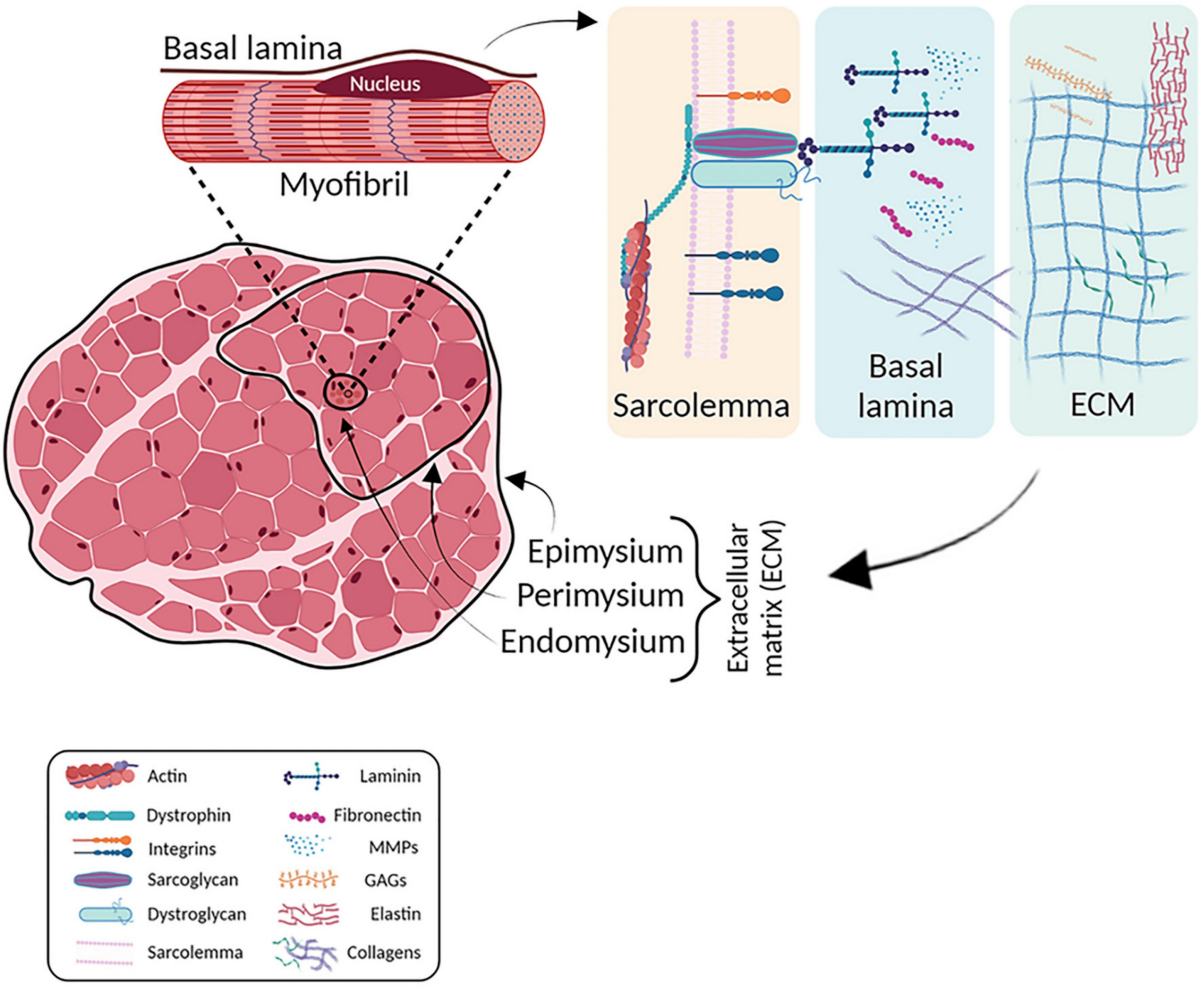
Schematic representation from micro to macro of muscle structure MMPs: matrix metalloproteinases, GAGs: glycosaminoglycans Image Credit: Adapted from Carraro et al. [26] with permission (Carraro, personal communication).

Figure 1 presents the representative diagram from the literature [26], illustrating the transmission of tension generated at the sarcomeric level, thus at the microscopic level, to the epimysium, the outermost fascial component of the muscle.

The basal lamina envelops each muscle fiber in contact with the sarcolemma, helps maintain the integrity and stability of the muscle fiber, and also separates and facilitates adhesion between the sarcolemma and the surrounding connective tissue, contributing to the transmission of mechanical forces during muscle contraction. Structurally, the basal lamina is an ECM composed primarily of proteins such as type IV collagen, laminin, and perlecan, which act as a scaffold for muscle cells and connect to the endomysium [27], which constitutes a three-dimensional network of channels within which muscle fibers operate.

Additionally, specialized connections have been identified [28] between myofibrils, their basal lamina, the endomysium, and the perimysium. Some authors propose that, structurally, the basal lamina can be conceptually included under the broader term endomysium [25,29].

Intramuscular connective tissue components

The organization of connective tissue within different types of muscle serves at least two fundamental roles: ensuring the proper alignment of muscle cells through their cohesion and enabling the transmission of force generated during muscle contraction [30].

Connective tissue has its own cellular population, represented mainly by fibroblasts. This connective tissue, known as the muscle ECM or intramuscular connective tissue (IMCT) [31], determines the spatial organization of skeletal muscle. IMCT is not limited to this organizational role but exhibits a specialized structural configuration at sites where the muscle connects to the aponeurosis and tendon [32].

Depending on its localization within the muscle, the connective tissue presents marked differences in the composition and distribution of IMCT molecules. These characteristics influence the degree of organization and flexibility, conferring specific functions to each compartment [17,33]. The overall mechanical properties of muscle depend both on the intrinsic characteristics of myofibrils and on those of IMCT, as well as their interaction. The close relationship between connective tissue and muscle tissue is a key element influencing muscle function [34].

It is incorrect to state that the endomysium and perimysium isolate or separate muscle fibers and fascicles as independent elements, since they actually create an interconnected three-dimensional network that also includes the epimysium [31]. This concept aligns perfectly with the observation that force transmission from contracting fibers occurs not only longitudinally toward the tendon but also laterally, between adjacent fibers and fascicles within a muscle [35,36].

The ECM not only plays a key role in maintaining the structure and organization of myofibers and transmitting force among muscle components but also ensures effective regeneration following injury [18,37,38]. Furthermore, the ECM generates biochemical signals that regulate myogenesis and modulate various growth factors [39-41].

These connective pathways are capable of transmitting force produced by myofibrils not only between isolated fibers but also within fascicles and throughout the whole muscle dissected in situ [42,43]. This mechanism of force transmission through IMCT [44] implies that muscle fibers cannot be considered isolated functional units but rather as integrated parts of a myofascial unit.

In clinical practice, however, the concept of muscular IMCT is often simplified: it is commonly understood as the connective structure surrounding the muscle, fundamental to ensuring its mechanical integrity. This connective structure is also sometimes generically referred to as “fascia” [45].

**Figure 2:**
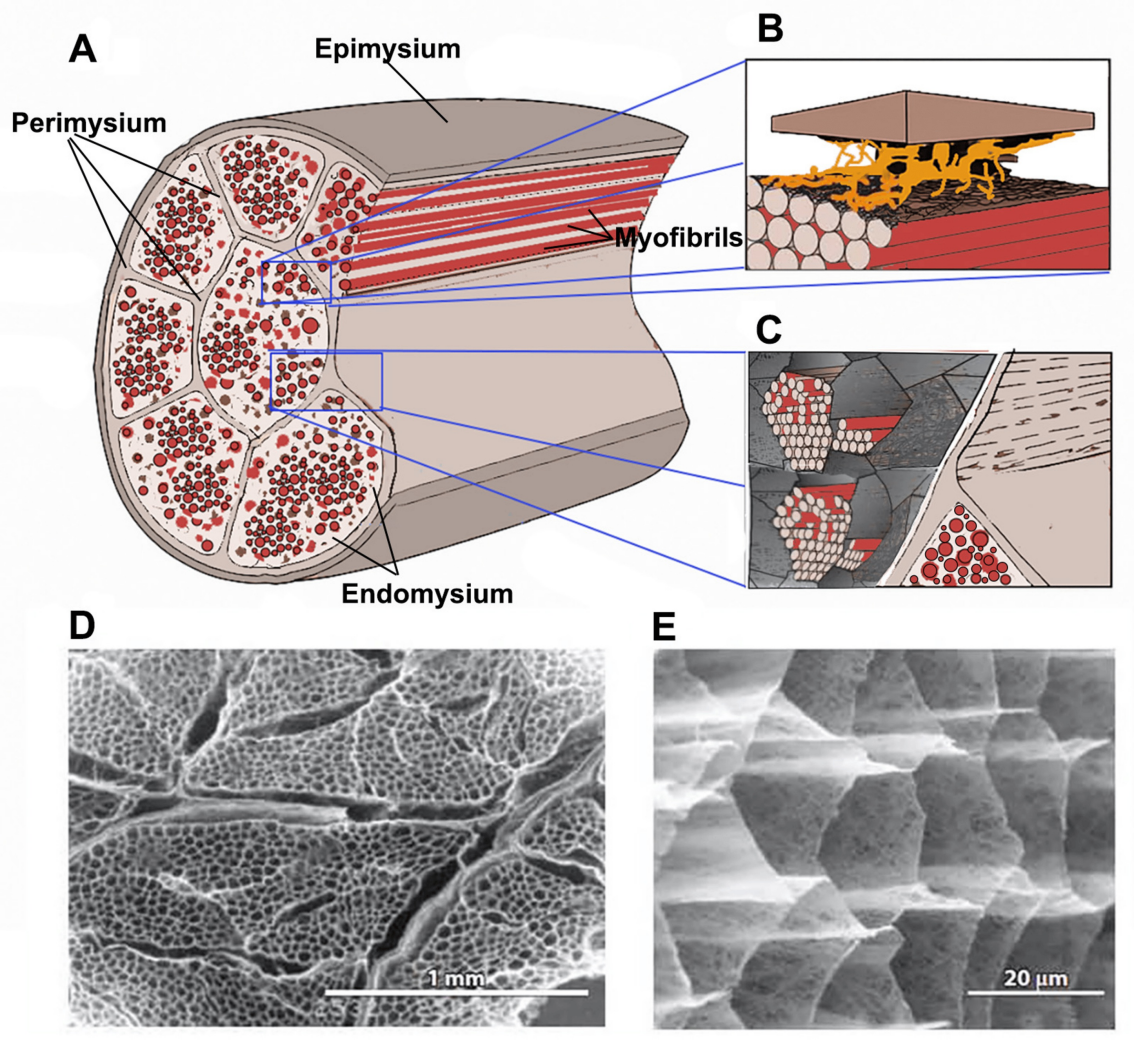
General structure of IMCT (A) Diagram illustrating the general arrangement of the epimysium, perimysium, endomysium, and myofibers within the muscle. (B) Diagram showing the rare junctional areas between the thick perimysium and the endomysium of muscle fibers in the superficial layer of the fascicle. (C) Diagram highlighting the myofibers of a single muscle cell embedded within the honeycomb-like endomysial network. (D) Low-magnification scanning electron micrograph of IMCT structures after treatment with NaOH to remove myofiber proteins and proteoglycans. The thick perimysium surrounding the honeycomb endomysial network within a fascicle is visible. (E) A higher magnification image of the endomysial network after NaOH treatment. IMCT: intramuscular connective tissue, NaOH: sodium hydroxide Image Credit: Saverio Colonna. Panels A, B, and C are original illustrations inspired by the model proposed in Purslow [31]. Panels D and E are reproduced from Trotter and Purslow [48] with permission (license to reuse obtained via CCC with order confirmation).

The collagenous fiber networks within the muscle are organized into three distinct layers within the muscle belly: endomysium, perimysium, and epimysium [18,45]. The endomysium surrounds each myofiber and is composed predominantly of type I, III, and V collagen [46], with fiber diameters of approximately 100-120 nm [34]. Situated between the basal laminae of adjacent muscle fibers, the reticular layer of the endomysium constitutes a continuum connecting these membranes. Its thickness has been estimated to range between 0.2 and 1.0 μm [47]. The thin collagen fibers that form its main component, embedded in a proteoglycan-rich amorphous matrix, create a planar network of wavy fibers with an almost random arrangement, as shown in Figure 2, reported by Purslow [31,48].

Transmission electron micrographs of transverse endomysial sections show that all collagen fibers lie in a plane parallel to the surface of the muscle fibers [49]. The preferred orientation of collagen fibers in the endomysial network varies according to the sarcomere length of the muscle. Still, it remains predominantly wavy regardless [48], thereby allowing relative compliance under tensile loading.

The perimysium surrounds bundles of myofibers and is composed mainly of type I and III collagen fibers with diameters of 600-1800 nm [34]. It should also be noted that the elastic protein elastin is present in small amounts in the perimysium of most muscles but is significantly increased in muscles such as the latissimus dorsi and semitendinosus of cattle [50], where it is believed to act as a reservoir of elastic energy. Elastin fibers are predominantly associated with the perimysium and epimysium of bovine semitendinosus and longissimus dorsi muscles [51].

The epimysium, which envelops the entire muscle, is also composed mainly of type I and III collagen. Additionally, type IV collagen is localized primarily in the basal membrane of the myofiber [19]. In pennate muscles, the thicker epimysium is organized into a laminar aponeurotic-like structure, which serves as a broad base for muscle insertion [52].

These three connective structures constitute what is defined as the myofascial junction (MFJ), representing the interface between the myofiber and fascia, or the MTJ, representing the connection between muscle and tendon. Both junctions have a structural function and contribute to the mechanical transmission of force [14].

From our perspective, the term "myotendinous" is misleading because histologically, it appears to describe the junction between muscle tissue and connective tissue. Both are derived from the mesoderm but originate from distinct subpopulations and developmental programs [53], overlooking, as discussed, their actual nature, namely, the junction between IMCT and tendinous connective tissue.

Aponeurosis

Compared to skeletal muscle and free tendon, the aponeurosis is a less extensively studied structure in the scientific literature, despite representing a substantial fibrous connective element that serves as a link between muscle and tendon. Furthermore, the terminology used in studies is highly heterogeneous: the aponeurosis is sometimes referred to with different terms such as “central tendon,” “intramuscular tendon,” “spine,” “septum,” or “inter-tendinous” [54-57].

In numerous studies, the aponeurosis is often equated with the free tendon or is not considered a distinct and autonomous structure. However, this assumption may not be correct, as in the muscle-aponeurosis-tendon model, the aponeurosis and tendon likely do not act as elements mechanically arranged strictly in series [58]. In fact, given its complex three-dimensional organization, the aponeurosis may not readily fit into either a “series” or “parallel” classification [58,59]. Moreover, the aponeurotic structure shows considerable variability among different muscle-tendon units, a characteristic that could influence both its mechanical role and its adaptive capacity.

Muscle-tendon unit as a connective continuum between intramuscular and tendinous tissues

The complex formed by a muscle and its associated connective tissues is known as the MTU. The function of this unit depends heavily on the interaction among its components (skeletal muscle, tendon, and aponeurosis), whose integration is essential for the execution of complex motor activities such as walking, running, and jumping [60,61]. Although MTJ is widely used in the literature [62], it is essential to acknowledge that alternative interpretations exist regarding the definition and structural features of this interface [3,13,14,16]. In many muscle-tendon units, it would be more accurate to refer to a myoaponeurotic junction, since the transition point often consists of the contact between muscle fibers and the aponeurosis.

**Figure 3:**
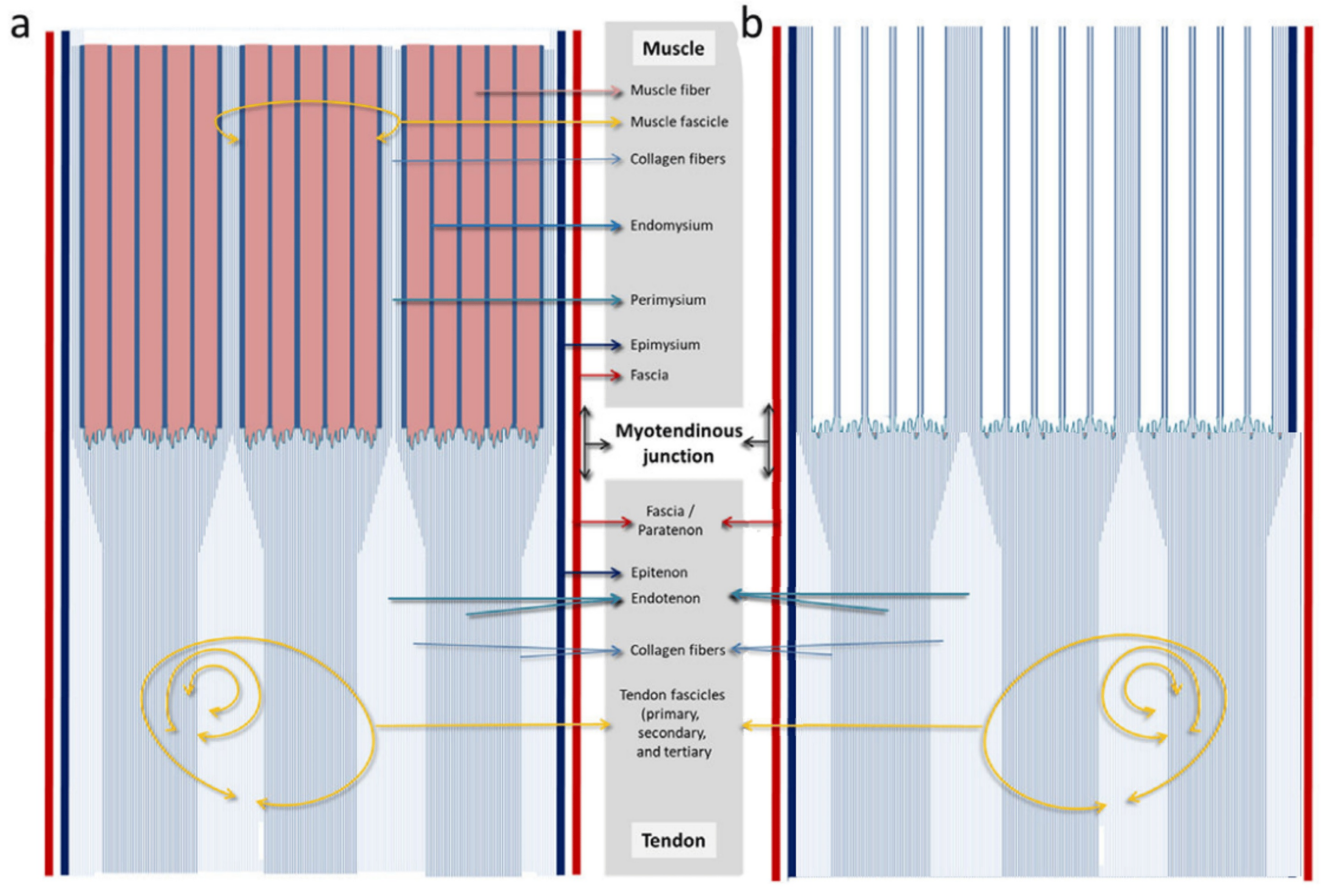
Diagram of the structural continuity between muscle and tendon (a) The muscle fascia continues with the tendinous fascia (or paratenon) and the epimysium with the epitenon. We have highlighted how the endomysium ends right at the muscle fibers, and the perimysium, at the end of the fascicle, continues to penetrate the tendon and form part of it. (b) The muscle fibers have been removed, and the highlighting of the endomysium and perimysium therefore shows the continuity of the musculotendinous connective frameworks with greater clarity. Image Credit: Adapted from Peña-Amaro [29] with permission (Peña-Amaro, personal communication).

Morphologically, the MTU appears as a series of connective folds resembling ridges that extend into the muscle tissue (Figure 3). These structures penetrate the muscle fiber membrane and project toward the myofibers, forming a complex and articulated interface [63].

The viscoelastic nature of the MTU enables efficient regulation of the relationship between force production, changes in unit length, and contraction velocity [64]. Moreover, the MTU exhibits remarkable adaptability, as its structure can be altered by various mechanical stimuli, including training [65], trauma [66,67], periods of immobilization [68,69], or aging [66,68].

Among the components of the MTU, skeletal muscle is certainly the most studied [18,70]; however, its passive properties remain less explored compared to its active characteristics [71]. The free tendon represents another fundamental component of the MTU and has been extensively studied for its passive ability to store elastic potential energy [72-75].

Injuries involving the MTU often entail damage or alteration of the aponeurotic tissue; however, the literature primarily focuses on muscle and tendon. As a result, aponeurotic injuries are frequently misinterpreted. For instance, many studies refer to injuries located at the "muscle-tendon junction" without acknowledging that the free tendon connects to the muscle through a complex network comprising the aponeurosis and ECM [14,76].

Aponeurotic tissue is often involved, with variable severity and localization along the longitudinal axis of the myoaponeurotic complex [49,51,61,63,64], especially in association with acute stretch-induced muscle injuries [45,60,65-67]. A recent consensus statement emphasized the importance of precisely distinguishing both the type and location of injuries affecting the myoaponeurotic junction, as these factors may have significant implications for prognosis and recurrence risk [61].

The tendon connects to the muscle via the MTJ and extends through the fascial layers and walls into the muscle. One of the most evident features in both tendon and muscle is the high degree of alignment in their collagen fibers, which serve as primary structural support elements in both tissues. This suggests a logical continuity between the two connective structures [29].

As the perimysium approaches the muscle surface, it merges seamlessly with the epimysium [77]. At the muscle extremities, the epimysium thickens and blends with tendinous structures [78]. This continuity relationship suggests that the epimysium, connected to the perimysium, acts as a superficial tendon to assist in transferring force from the muscle fiber to the skeleton. Accordingly, the fascia surrounding the epimysium continues into the fascia or paratenon (if present), which envelops the epitenon (Figure 3) [29].

Connective connections have been observed between different muscles and the deep fascia of limb muscles, suggesting that these connections provide myofascial continuity among the various limb muscles. It has been hypothesized that this continuity between IMCT and fascia coordinates the action of agonist muscles [79].

Within this hierarchy of connections, the nature of the links between the endomysial and perimysial networks on the surface of muscle fascicles is less well defined and requires further investigation. The perimysium typically continues directly into tendon subunits. One structural organization of perimysium in muscle is the honeycomb-like tube arrangement that connects the two tendons; the ends of these perimysial tubes form tendons and aponeuroses. Thus, collagen fibers of the perimysium penetrate directly into the tendon, becoming an integral part of it [18].

This implies that when referring specifically to the region of the myotendinous or myoaponeurotic junction, where the ends of muscle fibers anchor to collagen fibers, we are effectively located in the territory of the perimysium [29]. Not the perimysium that envelops or groups muscle fibers, but rather its terminal (more or less fusiform) region of the fascicle, where collagen fibers of the perimysium penetrate the tendinous regions [29]. Although both the endomysium and endotenon mainly contain type IV and type VI collagen as structural components, there is no direct continuity between them [29].

In any case, while we attempt to schematize this complex system, as illustrated in Figure 3, we are aware that the organization and orientation of collagen fibers are far more complex in both muscle and tendon. It is important to remember that both the quantity and morphological distribution of IMCT are highly variable among muscles with different functions [31], just as there is variability in the shape, size, and position (intra- or extramuscular) of tendons [55]. This variability may complicate or hinder the diagnosis of injuries occurring in these regions in a sports setting. In a stretch-induced muscle injury, the affected site always involves a junction composed of connective tissue, whether at the myotendinous or myofascial level [13]. Therefore, characterizing and systematizing the different histoarchitectural patterns in the muscle-tendon relationship is essential for classification [80] and familiarity with the clinical features, diagnostic strategies, therapeutic protocols, and prognostic outcomes of different injury types [13,14].

Nonetheless, it is surprisingly evident how little is known about the properties and adaptability of IMCT compared to the understanding of muscle function and plasticity deficiencies already highlighted in 2004 by Kjaer [35], with few advancements since then.

Tendon

From a hierarchical perspective, the tendon exhibits a histological and architectural organization similar to that of skeletal muscle. However, there is no clear terminological or structural equivalence with IMCT.

The tendon is externally covered along its entire length by the epitenon, a layer of loose connective tissue that serves as a smooth gliding surface for the tendon fascicles and also provides supply routes for the nervous and lymphatic systems of the tendon.

**Figure 4:**
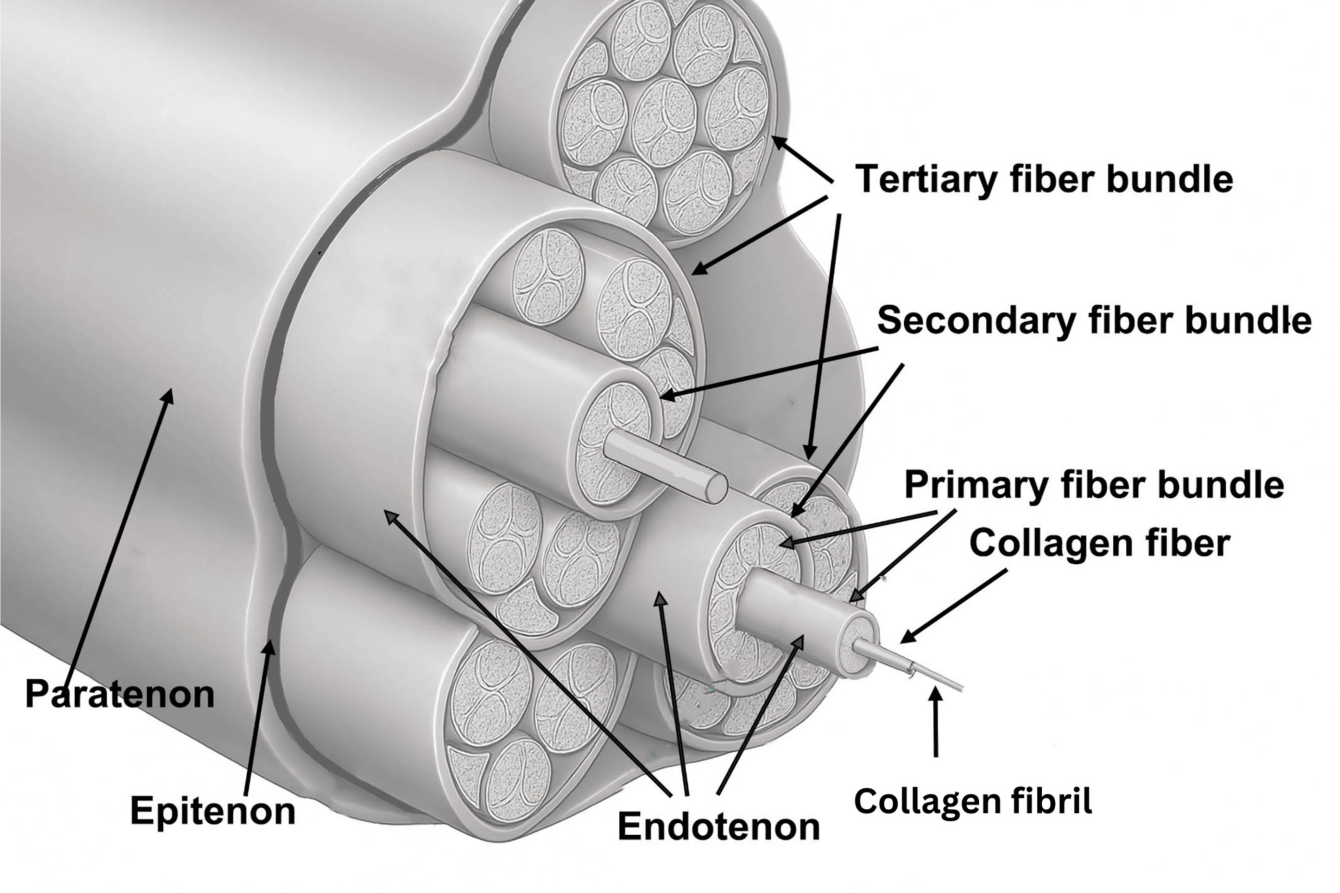
Schematic representation of tendon structure The picture shows the subdivision into primary, secondary, and tertiary subunits, along with the organization of the endotenon, epitenon, and paratenon. Image Credit: Saverio Colonna. Original figure created by the author; no permission required.

Internally, the endotenon (which is the basal membrane containing type IV and VI collagen) surrounds each tendon fiber and also links individual fibers into larger units that form the tendon fascicle (Figure 4). Toward the outside, the endotenon continues into the epitenon.

Another layer that some tendons may have is the paratenon, a synovial sheath that surrounds the epitenon, and together they are referred to as the peritenon [81]. The function of the paratenon is to provide lubrication to cushion the tendon and reduce friction with adjacent tissues as the tendon elongates and relaxes [82].

Tendons are also externally enveloped by fascia; the deep fascia, in addition to surrounding the muscle, also surrounds the tendons, forming a medium around both [78]. According to some authors [29], this means that the paratenon and the sheath may be the same structure: both are observed on ultrasound as a hyperechoic line [83], and, when this line is present for one of them, the other is absent [84]. The difference lies in the fact that while the paratenon displays a synovial sheath between itself and the epitenon, the sheath would be separated from the epitenon by loose connective tissue.

Distally, tendons attach to bone through the enthesis, a region characterized by a gradual transition from tendon to fibrocartilage to mineralized fibrocartilage and finally to bone, providing a smooth gradient of stiffness that reduces injury risk under high loads [85,86].

Musculoskeletal system: an advanced engineering system

**Figure 5:**
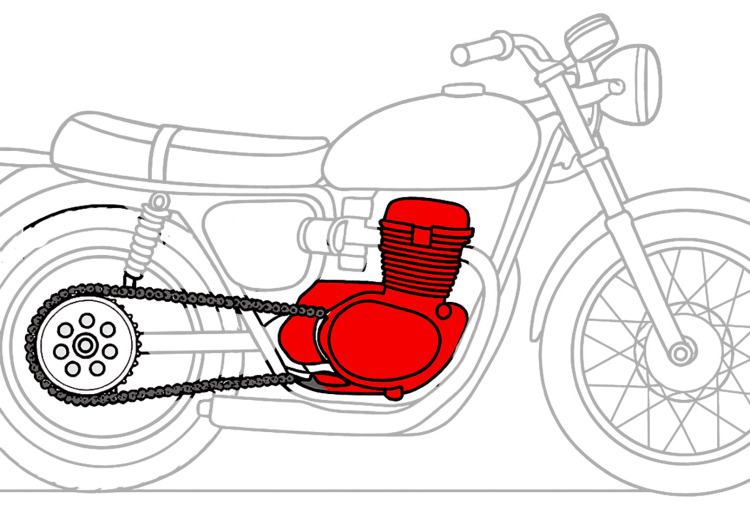
Schematic representation of a mechanical locomotor system (internal combustion engine) used as an analogy for the human musculoskeletal system Diagram illustrating the analogy between a muscle and a motorcycle, where the engine is represented by the myofiber and the transmission chain by the connective system. Image Credit: Author Saverio Colonna. Original figure created by the author; no permission required.

The musculoskeletal system can be compared to a locomotor system, such as that of a motorcycle or a car: the myofiber represents the engine, where force production occurs through energy consumption; the connective system (fascia, aponeuroses, tendons) functions like the transmission chain, which transfers the generated force (Figure 5); finally, the bone acts as the wheel, transforming the chain tension into movement.

All of this is coordinated by the control unit, which is the central nervous system. An even more fitting analogy would be with the hybrid locomotion systems currently in use. In these systems, part of the engine generates direct mechanical energy by burning fuel. In contrast, another part recovers energy, storing it as potential energy (e.g., in batteries), which is then utilized by electric motors to enhance the system's power output.

As previously stated, there is extensive research and knowledge regarding what happens within the motorcycle engine; however, there is limited research and understanding of the chain system that transmits the engine’s action to the wheels. In two recent articles [87,88], we reported how this transmission system includes receptor systems that detect tightness and how, using these data, active systems can adapt the tension of these “chains” to the required function.

Biomechanical studies on models and in vivo estimate that over 30% of mechanical energy during gait is stored and passively returned by elastic structures (tendons and IMCT), thereby reducing the active metabolic load of the muscles [60,72,89]. Elastic materials are those that display spring-like properties. When subjected to loading, they accumulate strain energy due to modifications in molecular bonds and conformational changes in the tertiary or quaternary protein structure.

In the case of tendons and ligaments, this behavior primarily results from the elongation of collagen fibers. In muscle, however, other elastic proteins also play a role, such as those in the cross-bridges (myosin I) and within the sarcomere (particularly titin), in addition to collagen itself.

These structures constitute the parallel and series elastic elements of the muscle, connected to the tendon, which represents the ultimate series elastic element [90]. During unloading, the stored elastic energy is released and can contribute to the mechanical movement of the body or a body segment, thereby reducing the workload required from the muscles.

Muscle work corresponds to the product of the force developed and the change in muscle length: shortening equates to positive work, whereas lengthening corresponds to negative work (i.e., energy absorption). In purely elastic elements, all energy accumulated during loading is returned during unloading. However, biological materials exhibit nonlinear elasticity and a certain degree of viscoelastic energy dissipation in the form of heat [91]. For this reason, materials such as tendons are defined as viscoelastic.

The percentage of elastic energy determines the resilience of a material, effectively recovered relative to that stored. In tendons, this resilience ranges between 90% and 94%, meaning that only 6-10% of the accumulated energy is dissipated as heat in each cycle of muscle contraction [91].

During muscle stretching, intramuscular connective structures, such as the aponeurosis, significantly contribute to muscle mechanics. Experimental studies [92] conducted on the rat medial gastrocnemius demonstrated that the aponeurosis elongates more under passive conditions than active ones, reaching extensions of up to 10% when the muscle is stretched from slack length to optimal length. In contrast, the same stretch under electrical activation produces less aponeurotic elongation at the same muscle length.

During passive stretching, the aponeurosis proves significantly more extensible and better suited for elastic energy storage compared to elongation induced by active muscle contraction, because muscle contraction induces biaxial loading that increases its stiffness [93].

This behavior suggests that, under passive conditions, the connective component exhibits greater compliance and thus a higher capacity for storing elastic energy. In other words, the potential energy stored as elastic deformation is greater during passive movement compared to active contraction, where the overall stiffness of the system limits connective tissue deformability.

These findings support the hypothesis that passive stretching phases may represent a critical moment for mechanical overload of parallel fascial structures, including the aponeurosis [94,95]. Moreover, the study reports that aponeurosis extension is distributed heterogeneously along its length. The extensions observed for the aponeurosis were greater in both terminal regions compared to intermediate regions, consistent with strain measurements in tendons and fascia [96]. Variations in aponeurosis extension may be caused by a non-uniform distribution of force and/or differences in compliance along the aponeurosis.

Furthermore, the degree of muscle injury is correlated with the extent of passive stretch during contraction [97]. High shear stress at the interface between myofiber and the endomysium could result in damage to the sarcolemma, the basal lamina, and the dystrophin-associated glycoprotein [97].

Role of the myofascial elastic component

Evidence that the passive elastic component of the leg is important for gait and running comes from studies on leg amputees, where the use of specialized springs, purely passive structures capable of reusing energy, can effectively, or nearly completely, replace the active muscular structures of the calf [98,99].

In muscle, a mechanism similar to that described above for hybrid cars/motorcycles occurs due to the ability to reuse part of the mechanical energy produced. During the elongation of certain viscoelastic tissues, such as intra- and perimuscular connective structures, potential elastic energy is stored. This energy can subsequently be released in the form of kinetic energy, actively contributing to the total mechanical force production of the muscle. This model reduces metabolic cost, fatigue, and potentially muscle damage [72].

Pennate muscles

In the musculoskeletal system, pennate muscles appear to exemplify this model particularly well. Pennate architecture is especially prevalent in the muscles of the lower limbs, such as the gastrocnemius, soleus, and vastus muscles, compared to those of the upper limbs and trunk. This distribution may be interpreted as a functional adaptation aimed at optimizing force production and elastic energy recovery during repetitive, cyclical activities like gait [90].

During the eccentric phase of the gait cycle (stance and loading), muscle elongation occurs due to gravitational forces and leads to stretching of viscoelastic tissues (such as aponeuroses and tendons), which store potential energy. This stored energy is subsequently reused during the concentric phase to aid in movement generation, thereby enhancing the energy efficiency of the musculoskeletal system. Muscle pennation reduces direct fiber elongation and increases the contribution of connective tissues, promoting the storage and release of elastic energy [61,100].

By contrast, muscles of the upper limb or trunk, typically fusiform or with minimal pennation, serve purposes more related to mobility, precision, and control, rather than sustained support and efficient locomotion [101]. Thus, the pennate arrangement in lower limb muscles may represent an evolutionary architectural strategy designed to maximize human movement efficiency by reducing metabolic expenditure during repetitive locomotor activities [102-105].

A study by Azizi and Roberts [100], for example, used sonomicrometry to analyze in vivo the functional differences between the pennate-fibered lateral gastrocnemius and the parallel-fibered sartorius muscle. The results showed that in pennate muscles, most of the shortening of the whole muscle was due to deformation of the aponeurosis, while the muscle fibers themselves shortened relatively little. In contrast, in parallel-fibered muscles, fiber shortening was more directly correlated with the movement of the whole muscle.

This study highlights that the interaction between the contractile unit and connective tissues (such as the aponeurosis) varies according to muscle function and that in locomotor muscles, the fascial system plays a key role in elastic energy recovery and transmission.

Aponeurotic injuries

Returning to microscopic anatomical structure, aponeuroses exhibit direct connective continuity with the MFJ through the perimysium [18]. From a histological perspective, as previously described, the perimysium connects to the aponeurosis at the ends of muscle fascicles, thereby contributing to the formation of an integrated functional unit between the muscle fascicles and tendons within skeletal muscle [13].

In every indirect trauma-related “muscle injury,” i.e., stretch injury, a myoconnective junction is almost always involved [13]. This may occur at the MTJ when the injury involves an aponeurosis or a tendinous expansion, or at the MFJ when the muscle fiber is connected to the epimysium or perimysium [13].

Muscle aponeurotic injuries can be conceptualized as peripheral or central [14]. Peripheral injuries involve aponeuroses located on the muscle surface, such as the distal aponeurosis of the long head of the BFlh [106], the posterior aponeurosis of the rectus femoris [107], and the anterior aponeurosis of the gastrocnemius [76]. Central injuries, on the other hand, affect intramuscular aponeuroses that are surrounded by muscle fascicles and perimysium but not by epimysium [14].

Damage may involve entirely intramuscular aponeuroses, such as those of the soleus [108] or rectus femoris [15], or partially intramuscular aponeuroses, such as the proximal intramuscular portion of the BFlh aponeurosis [54,109]. Some myoaponeurotic injuries are direct and involve only the aponeurotic tissue [3,14]. The direction of the injury can be transverse, longitudinal (i.e., “splitting”), or mixed [14].

The orientation of the injury is clinically relevant: for example, transverse injuries cause muscle retraction, whereas isolated longitudinal injuries usually do not [14]. It has also been hypothesized that longitudinal injuries carry a higher risk of recurrence, as they tend to heal with less fibrous tissue formation [14]. However, other studies have shown that even transverse or mixed injuries, when associated with a transverse disruption of aponeurotic continuity visible on MRI or scar tissue formation, may be linked to a high risk of recurrence [110].

Other myoaponeurotic injuries involve the junction between the aponeurosis and muscle fibers together with their perimysium [3,14]. Even a small muscle injury can produce a focal aponeurotic disruption during the coalescence phase. In more severe cases, significant muscle injury can cause detachment of the epimysium from its aponeurosis, creating an anatomical “gap” while the aponeurosis remains intact [14,76].

The connection between aponeurosis and myofibers is never truly “direct” in the strict histological sense. Still, it is always mediated by connective structures, particularly the endomysium, which envelops each muscle fiber and gradually integrates with the perimysium and then the aponeurosis, a dense connective lamina similar to a tendon [31,111], as part of the complex structure known as the ECM [18,31,112].

For this reason, some authors have concluded that connective tissue integrity can be used to estimate and guide return-to-play timing in cases of calf “muscle injuries” [113]. As proposed by Blasius and his study group (Study Group of the Muscle and Tendon System) [13], muscle injuries always involve a connective junction. This may occur at the MTJ, when the injury affects an aponeurosis or tendinous expansion connected to the muscle fibers, or at an MFJ, when the muscle fibers are anchored to the epimysium or perimysium.

Active or passive muscle component injury?

Indeed, a “muscle injury” occurs within the muscle, but which histological component of the muscle is truly affected? The histological structure of the muscle consists of an active part responsible for producing muscle tension, primarily constituted by muscle fibers, and a passive part responsible for transmitting the produced tension, composed of the connective tissue system [31].

A recent consensus statement emphasized the importance of precisely distinguishing both the type and the location of injuries affecting the myoaponeurotic junction, as these factors can significantly influence prognosis and recurrence risk [14].

Identifying the functional role of the different structures that comprise “muscle” allows for a better understanding of dysfunctions relevant to the prevention and treatment of stretch-related injuries. The answer could prompt a revision of the principles underlying the prevention and therapy of these injuries. Indeed, many preventive and therapeutic exercise methods are based on models that primarily target the contractile component of the muscle, i.e., force production [114,115].

Several systematic reviews report that progressive programs, which include eccentric stretching exercises combined with trunk stabilization and agility drills, significantly reduce return-to-sport time and recurrence risk [115,116]. According to clinical guidelines [115], the early phases of hamstring injury rehabilitation should include trunk isometric exercises, single-leg balance training, and short-step frontal-plane drills.

To date, few studies have directly investigated the effect of resistance training (RT) on connective tissue adaptations. Available studies have reported increases in both size and strength of tendons and ligaments [114]. It has been shown that tendons and ligaments respond to RT with increased metabolism, thickness, and strength [117]. Additionally, research indicates that damaged tendons and ligaments regain strength more rapidly when RT is performed after injury [117]. Furthermore, although collagen content increases with training, comparisons between untrained individuals and bodybuilders suggest that such increases are proportional to muscle growth. Therefore, muscle hypertrophy is likely accompanied by increases in connective tissue size and strength [114], resulting in improved tensile resistance. For further details on the fascial component’s response to exercise, we refer to our previous articles [87,88].

Previous studies suggest that one of the main benefits of RT lies in learning to coordinate the different muscle groups involved in the exercise movement, rather than solely increasing intrinsic strength in the specifically trained muscle group [118]. This implies that well-coordinated muscles can decelerate joint movements smoothly even if their strength is relatively low. The most common cause of impaired neuromotor coordination is prior injury. As a protective mechanism against further damage, the central nervous system establishes an alternative muscle recruitment pattern, defined as a motor engram, to avoid stressing the injured soft tissues [119]. If proper rehabilitation is not undertaken, this motor engram persists even after injury healing, predisposing individuals to recurrences. In this context, RT has been shown to increase motoneuron excitability and induce synaptogenesis, thereby improving communication between the nervous system and the muscles [114].

The initial question thus aims to prompt reconsideration of which component, in reality, is injured. Moreover, there is a lack of knowledge regarding the assessment of the health status of the intramuscular fascial system, which constitutes the primary component for tension transmission. The only study, to our knowledge, that has addressed this topic is the systematic review by Wilk et al. [120], which in its conclusions states:

The present systematic review is the first study to summarize the evidence on the prevalence of fascial lesions in clinically diagnosed muscle strain injuries. In both sports practice and scientific research, it has been widely assumed that strains occurring in the soft tissue predominantly affect the skeletal muscles. Our findings contradict this assumption; isolated muscular lesions were identified only in about 1 of 8 cases, and the damage was frequently located within or at the junction to the collagenous connective tissue. The term “muscle strain injury,” therefore, does not adequately reflect the morphological substrate of the condition and could be misleading during the diagnostic process. To avoid this, we suggest using more general terms (eg, “myocollagenous strain injury”) that may indicate more clearly the variety of potentially affected tissues.”

The aponeurotic tissue, therefore, is frequently compromised, with varying degrees of severity and localization along the longitudinal axis of the myoaponeurotic complex [13,14,76,121], either in association with or isolated from acute muscle strain injuries [14,54,110].

To facilitate understanding of the topics addressed, Table 2 summarizes the key structural and functional concepts of muscle injuries together with their diagnostic and rehabilitative implications.

**Table 2:** Summary of key concepts in muscle injuries The table summarizes the main themes discussed in the review, providing a conceptual overview from microstructural organization to clinical implications. IMCT: intramuscular connective tissue, MTJ: myotendinous junction

Topic	Key insights	Clinical/diagnostic/rehabilitation implications
IMCT (endomysium, perimysium, epimysium)	Provides structural integrity and transmits force both longitudinally and laterally	Highlight the role of connective tissue in diagnosis and injury prevention
Myoaponeurotic junctions and MTJ	Frequent sites of stretch-related injuries and potential weak links	Imaging and prognosis should consider the specific junction involved
Aponeuroses	Not simple extensions of tendons; heterogeneous mechanical behavior	Rehabilitation should target connective as well as contractile tissue
Elastic energy storage (tendon + fascial system)	Significant proportion of mechanical energy stored and released by elastic structures	Exercise programs should address both strength and elastic recovery
Classification systems	Different frameworks (mechanism-, clinical-, or imaging-based) provide complementary perspectives	Useful to tailor prognosis, guide therapy, and reduce recurrence risk

## Conclusions

A comprehensive understanding of muscle injuries requires a shift from a purely contractile perspective toward an integrated model that includes the myoconnective architecture. Skeletal muscle is not merely a collection of myofibers; it is a complex composite in which the IMCT (endomysium, perimysium, and epimysium), basal lamina, aponeuroses, and the ECM all contribute to mechanical performance and injury susceptibility. This structural continuum transmits force not only longitudinally through tendons but also laterally across adjacent fibers and fascicles via the IMCT network, supporting both stability and coordination under dynamic loading.

The recognition that indirect muscle injuries often involve myoconnective junctions, whether myotendinous, myoaponeurotic, or myofascial, highlights the relevance of these viscoelastic and mechanically critical interfaces. These zones of high stress concentration represent frequent sites of injury and may constitute the "weak links" of the musculoskeletal chain. Consequently, effective prevention and rehabilitation strategies must go beyond a myofiber-centric view and target connective tissue adaptations, elastic energy dynamics, and neuromuscular coordination patterns. By linking microstructural organization with macroscopic injury patterns, clinicians and researchers can better understand how contractile and connective components interact as a functional unit. This paradigm shift enhances diagnostic precision and informs rehabilitation strategies that address both active and passive tissue components.

In Part II, this integrated framework will be applied to the medial gastrocnemius lesion (“tennis leg”) as a prototypical example of how myoconnective architecture governs injury mechanics and guides clinical management in high-demand athletic populations.
